# Complement activation in association with clinical outcomes in ST-elevation myocardial infarction

**DOI:** 10.1016/j.ahjo.2022.100228

**Published:** 2022-11-19

**Authors:** Karsten E. Kluge, Miriam S. Langseth, Geir Ø. Andersen, Sigrun Halvorsen, Trine B. Opstad, Harald Arnesen, Theis Tønnessen, Ingebjørg Seljeflot, Ragnhild Helseth

**Affiliations:** a Center for Clinical Heart Research, Department of Cardiology, Oslo University Hospital Ullevål, Norway; b University of Oslo, Norway; c Department of Cardiology, Oslo University Hospital Ullevål, Norway; d Department of Cardiothoracic Surgery, Oslo University Hospital, Norway

**Keywords:** Complement system, Neutrophil extracellular traps, NETs, Coronary artery disease, ST-elevation myocardial infarction

## Abstract

**Introduction:**

The complement system and neutrophil extracellular traps (NETs) might contribute to ischemia-reperfusion injury in ST-elevation myocardial infarction (STEMI). We aimed to estimate associations between complement activation and NETs in STEMI, and their prognostic value on clinical endpoints.

**Methods:**

In this cohort study, 864 patients admitted for PCI during STEMI were included. Complement activation was analyzed by the terminal complement complex (TCC), while NETs were analyzed by myeloperoxidase-DNA, citrullinated histone 3 (CitH3) and dsDNA. The composite endpoint was reinfarction, unscheduled revascularization, stroke, hospitalization due to heart failure, or death, and the secondary endpoint was total mortality. The association between TCC and clinical endpoints was assessed by Cox regression and ROC curve analysis.

**Results:**

TCC was weakly correlated to dsDNA (*r* = 0.127, *p* < 0.001) and CitH3 (*r* = 0.102, *p* = 0.003). After a median follow-up time of 4.6 years, 184 (21.3 %) patients had reached a clinical endpoint. TCC was not associated with the composite endpoint, but with total mortality (HR: 1.673, 95 % CI: [1.014, 2.761], *p* = 0.044). The significant association was lost when adjusting for CRP, NT-proBNP, LVEF and time from symptoms to PCI. In ROC curve analysis of total mortality, the AUC for TCC alone was 0.549 (95 % CI: [0.472, 0.625]), AUC for dsDNA alone was 0.653 (95 % CI: [0.579, 0.720]), while AUC for TCC and dsDNA combined was 0.660 (95 % CI: [0.590, 0.730]).

**Conclusions:**

In this STEMI cohort, TCC was not associated with the composite endpoint, but somewhat with total mortality. Combining TCC and dsDNA did not increase the prognostic value compared to dsDNA alone.

## 1 Introduction

The complement system is an essential part of the innate immune system. Consisting of soluble and cell-bound proteins, it can be rapidly activated in response to pathogens or tissue damage [1]. The main function of the complement system is antimicrobial, but a role in the ischemia-reperfusion (IR)-injury following revascularization of hypoxic myocardium in ST-elevation myocardial infarction (STEMI) has been suggested [2]. IR-injury is estimated to cause up to 50 % of the myocardial damage following STEMI, yet its mechanisms are not fully understood. A central phenomenon is presumed to be an excessive immunological response [3], in which complement activation is thought to contribute through chemotaxis of inflammatory cells, inflammatory signaling in cardiomyocytes, as well as direct damage to cardiomyocytes through the terminal complement complex (TCC) [4], [5]. Complement activation measured by TCC has been reported to predict cardiovascular outcomes and death after STEMI and other types of myocardial infarctions [6], [7], and complement components have been detected in infarcted myocardium [8], [9], [10].

Another part of the immune system with emerging relevance in cardiovascular disease is neutrophil extracellular traps (NETs). These are structures consisting of nuclear material covered with neutrophilic granule proteins expelled from neutrophil granulocytes upon activation [11]. Several lines of evidence suggest that NETs contribute to the progression of atherosclerosis and have prothrombotic effects [12], [13]. Murine models support the contribution of NETs to IR-injury, as treatment with DNase, which dissolves NETs, reduces IR-injury [14], [15]. In human STEMI patients, NETs burden in coronary thrombi reflect infarct size and ST-segment resolution [16], while NETs markers in peripheral blood seem to reflect myocardial infarct size and predict clinical outcomes [17], [18].

Reciprocal activation of complement and NETs has been demonstrated experimentally [19], but it is not known if this happens during STEMI, and if so, how clinically relevant it is. Given that a reciprocal activation between complement and NETs has been hypothezised following revascularization in STEMI, our aim was to assess if there were associations between TCC and markers of NETs, whether TCC was predictive of clinical endpoints in patients with STEMI, and if combining TCC and NETs could predict clinical endpoints more precisely than TCC and NETs markers alone.

## 2 Materials and methods

### 2.1 Study population

Patients with STEMI admitted to Oslo University Hospital Ullevål, Oslo, Norway for percutaneous coronary intervention (PCI) were included between 2007 and 2011 (*n* = 1028). Patients were routinely included the first morning after the primary PCI procedure. Details of the study have previously been described [20]. Patients below 18 years old or patients unable or unwilling to give written informed consent were not included. Clinical information was collected from hospital records and questionnaires obtained at the time of inclusion. Left ventricular ejection fraction (LVEF) was assessed by echocardiography performed within three months after the index infarction, by either visual approximation or the Simpson's biplane method.

For the present sub-study, we excluded patients using oral anticoagulation due to its potential interactions with NETs, and a total of 864 patients were included. We have previously published data showing that the NETs marker dsDNA was associated with mortality in this population [18]. The study was approved by the Regional Ethics Committee of South East Norway (project ID 1.2006.1975).

### 2.2 Definitions

STEMI was defined as electrocardiographic ST segment elevation of >2 mm in two or more contiguous chest leads, >1 mm in two or more limb leads, or new onset of left bundle-branch block, together with chest pain or other typical symptoms and elevated troponin levels above the 99th percentile. Previous cardiovascular disease (CVD) was defined as previous myocardial infarction, ischemic stroke, PCI procedure, or coronary artery bypass surgery. Diabetes mellitus and hypertension was defined as treated diabetes and hypertension. Smoking was defined as current smoking or cessation <3 months prior to inclusion.

### 2.3 Laboratory methods

Blood samples were collected in fasting condition between 8:00 and 10:00 a.m. the morning after PCI as previously described [20]. For patients admitted during the weekend, inclusion was performed the following Monday morning. Collection was performed at a median time of 24 h after symptom debut and 18 h after PCI. Serum was prepared by centrifugation for 10 min at 2000 ×*g*, EDTA plasma was prepared by centrifugation for 30 min at 3000 ×*g* at 4 °C, and samples were stored at −80 °C until analyzed.

Plasma levels of the terminal complement complex (TCC) were quantified in plasma using a commercially available immunoassay (human TCC, HycultBiotech, Uden, The Netherlands). Results are presented as arbitrary units (AU), with an inter-assay coefficient of variation (CV) of 8.1 %. The NETs markers dsDNA and MPO-DNA were measured in serum as described elsewhere [18]. In short, dsDNA was quantified by a nucleic acid stain, Quant-iT PicoGreen (Invitrogen Ltd., Paisley, UK) and fluorometry (Fluoroskan Ascent fluorometer, Thermo Fisher Scientific, Vantaa, Finland), MPO-DNA was quantified by ELISA using the technique described by Kessenbrock et al. [21], with results reported as optical density units (OD), and citrullinated histone 3 (CitH3) was quantified by a commercially available ELISA (Cayman Chemical, Ann Arbor, USA). The intra-assay CVs were 6.3 % for dsDNA, 9.1 % for MPO-DNA and 12.4 % for CitH3.

### 2.4 Clinical endpoints

Patients were followed for a median of 4.6 years. The primary endpoint was a composite of reinfarction, stroke, unscheduled revascularization >3 months after the index infarction, rehospitalization for heart failure, or death from any cause, whichever occurred first. The secondary endpoint was total mortality during the follow-up period. Endpoints were collected by patient contact, hospital records and the Norwegian Cause of Death Registry, and were evaluated by an endpoint committee.

### 2.5 Statistical analyses

Data is presented as mean ± SD, median (25th, 75th percentile) or numbers (%) as appropriate. The unpaired Student *t*-test, Mann-Whitney *U* test and Kruskall-Wallis test were used to determine differences between groups as appropriate. Proportional data was compared using the chi-squared test. Correlation analyses were performed using Spearman's rho. Multivariate logistic regression was used to assess the predictive value of variables. Survival curves were generated using Cox regression, and crude and adjusted hazard ratios (HRs) were calculated using Cox proportional hazard regression models. Age and gender were included in the adjusted model by convention. Other covariates were included if they exhibited an association of *p* ≤ 0.10 with both TCC (Table 1) *and* the dependent variable. The receiver operator characteristic (ROC) curve analysis with the corresponding area under the curve (AUC) with 95 % confidence interval was performed to determine the predictive value of variables. *P*-values of ≤0.05 were considered statistically significant, and all statistical analyses were performed using IBM SPSS statistics v.27.

**Table 1:** Baseline characteristics of the total study population.

	Total study population (*n* = 864)	Above-median TCC (*n* = 432)	Below-median TCC (n = 432)	p-Value
Age, mean (range)	60.7 (24–94)	61.3 (31–91)	60.1 (24–94)	0.105
Female gender	173 (20)	92 (21.3)	81 (18.8)	0.350
Smoking	414 (47.9)	200 (46.4)	214 (49.7)	0.340
Hypertension	283 (32.8)	153 (35.4)	130 (30.1)	0.095
Diabetes	105 (12.2)	46 (10.6)	59 (13.7)	0.176
BMI, kg/m^2^	26.6 (24.3, 29.3)	26.6 (24.2, 29.3)	26.6 (24.5, 29.3)	0.609
eGFR	96.0 (86.2, 103.5)	95.8 (86.2, 103.3)	96.2 (86.0, 103.7)	0.713
Total leukocyte count × 10^9^/L	10.6 (8.65, 13.10)	10.8 (8.83, 13.0)	10.5 (8.50, 13.3)	0.521
Platelet count × 10^9^/L	219 (187, 264)	221 (188, 271)	218 (183, 260)	0.117
Total cholesterol, mmol/L	4.93 ± 1.94	5.04 ± 2.52	4.79 ± 1.12	**0.017**
LDL-cholesterol, mmol/L	3.25 ± 1.02	3.29 ± 1.01	3.20 ± 1.03	0.092
HDL-cholesterol, mmol/L	1.12 ± 0.40	1.13 ± 0.35	1.11 ± 0.45	0.144
Triglycerides, mmol/L	1.45 ± 0.88	1.40 ± 0.73	1.49 ± 1.00	0.840
Fasting glucose, mmol/L	5.8 (5.2, 6.6)	5.7 (5.2, 6.5)	5.8 (5.3, 6.7)	0.179
C-reactive protein, mg/L	13.39 (7.00, 31.22)	16.72 (8.86, 49.94)	10.67 (5.6, 22.09)	**<0.001**
Peak TnT, ng/L	3835 (1685, 7045)	4155 (1853, 7548)	3580 (1603, 6573)	0.085
NT-proBNP, pg/mL	31 (10, 116)	35 (12, 150)	24 (8, 84)	**<0.001**
LVEF ≤40 %	133 (15.4)	83 (24.6)	50 (17.6)	**0.002**
Symptom to PCI time, hours	4 (3, 6)	4 (3, 7)	4 (2, 6)	**0.004**
PCI to blood sampling, hours	18 (13,22)	19 (14, 25)	17 (11,21)	**<0.001**

Previous CVD				
Myocardial infarction	95 (11.0)	51 (11.8)	44 (10.2)	0.454
PCI	94	47 (10.9)	47 (10.9)	1.000
Heart failure	17 (2.0)	10 (2.3)	7 (1.6)	0.453
Stroke	37 (4.3)	20 (4.6)	17 (3.9)	0.614

Medication at hospital admission:				
Single or DAPT	195 (22.6)	97 (22.5)	98 (22.7)	0.935
Statins	190 (22.0)	89 (20.6)	101 (23.4)	0.324
Beta blockers	161 (18.6)	81 (18.8)	80 (18.5)	0.603
ACEi/ARB	206 (23.8)	109 (25.2)	97 (22.5)	0.376

Values are given as mean (±SD), median (25th, 75th percentiles) or numbers (%) as appropriate. BMI: body mass index; eGFR: estimated glomerular filtration rate; LDL: low-density lipoprotein; HDL: high-density lipoprotein; TnT: troponin T; NT-proBNP: NT-pro brain natriuretic peptide; LVEF: left ventricular ejection fraction; CVD: cardiovascular disease; PCI: percutaneous coronary intervention; DAPT: dual antiplatelet therapy; ACEi: angiotensin converting enzyme inhibitor; ARB: angiotensin II receptor blocker. Bold indicates p-value < 0.05.

## 3 Results

### 3.1 Study population

Baseline characteristics of the total population are shown in Table 1. In the total population, 20.0 % were women, and the mean age was 61 years. Almost half were smokers, 12.2 % had diabetes and 23.1 % had previous CVD. Median peak Troponin T (TnT) was 3835 ng/L, and 15.4 % had a left ventricular ejection fraction (LVEF) ≤ 40 %. Baseline characteristics according to endpoints are shown in Table S1.

### 3.2 Association between TCC, NETs and myocardial function

Both TCC and the markers of NETs exhibited a right-skewed distributed with median values: TCC 3200 AU (2762, 3941), dsDNA 410 ng/mL (370, 460), MPO-DNA 0.177 OD (0.139, 0.254) and CitH3 8.79 ng/mL (4.73, 16.60). TCC was significantly, but weakly, correlated to dsDNA and CitH3, but not to MPO-DNA, and there was a weak positive correlation between TCC, CRP, peak TnT and NT-proBNP (Table 2). Patients with a LVEF ≤40 % had significantly higher TCC than patients with LVEF >40 % (3445 AU vs. 3163 AU, *p* = 0.001).

**Table 2:** Correlations (Spearmans rho) between TCC, markers of NETs, CRP, peak TnT and NT-proBNP.

	dsDNA	MPO-DNA	CitH3	CRP	Peak TnT	NT-proBNP
TCC	r = 0.127 p < 0.001	r = 0.021 p = 0.543	r = 0.102 p = 0.003	r = 0.311 p < 0.001	r = 0.070 p = 0.039	r = 0.168 p < 0.001

TCC: terminal complement complex; dsDNA: double-stranded DNA; MPO-DNA: myeloperoxidase DNA; CitH3: Citrullinated histone 3; CRP: C-reactive protein; TnT: troponin T; NT-proBNP: NT-pro brain natriuretic peptide.

### 3.3 Association between TCC and clinical endpoints

During a median follow-up time of 4.6 years, 184 patients (21.3 %) reached the composite endpoint (55 deaths, 59 myocardial reinfarctions, 6 strokes, 51 unscheduled revascularizations >3 months after the index infarction and 13 hospitalizations for heart failure). In total, 70 (8.1 %) died during follow-up, with or without previously reaching the composite endpoint.

**Fig. 1:**
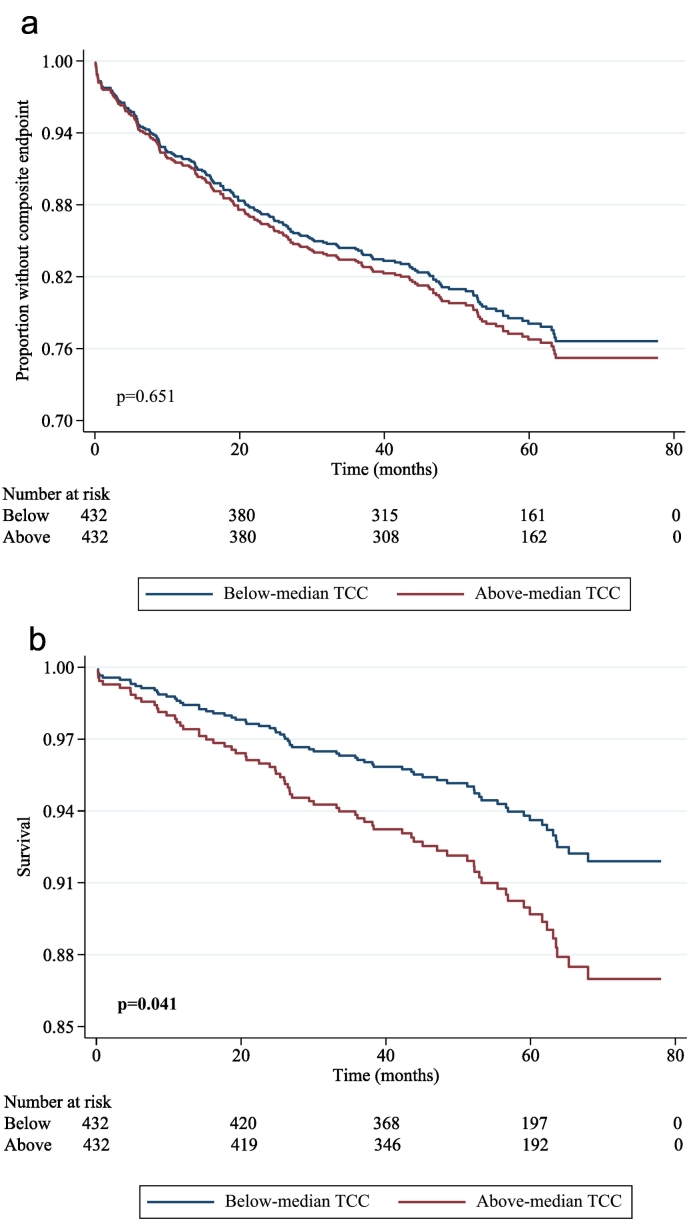
a. Survival free of events for patients with above vs. below median concentrations of TCC. TCC: terminal complement complex. b. Survival curve for patients with above vs. below median concentrations of TCC and total mortality. TCC: terminal complement complex.

When dividing TCC into quartiles, comparing the highest quartile to the lowest quartile, no increased risk of the composite endpoint was observed (HR 1.059, 95 % CI: [0.706, 1.586], *p* = 0.782, Fig. S1a). The same was found when comparing these quartiles and the risk of total mortality (HR 1.574 95 % CI: [0.836, 2.965], *p* = 0.160, Fig. S1b). When comparing the group with above-median TCC level to the group with below-median level in crude Cox regression, no increased risk of the composite endpoint was observed (HR: 1.069, 95 % CI: [0.801, 1.428], *p* = 0.651, Fig. 1a).

Patients with above-median TCC had a significantly higher rate of total mortality during follow-up (43 deaths vs. 27 deaths, *p* = 0.046). In unadjusted Cox regression, the above-median TCC group had an increased risk (Table 3, Fig. 1b), persisting after adjusting for age, gender, hypertension, and LDL cholesterol (Table 3, Model 1). With additional adjustment for CRP and NT-proBNP, the increased risk was no longer significant (Table 3, Model 2). This was also the case when adjusting for CRP and NT-proBNP separately (Table S2, Models 4 and 5). As we missed LVEF data on 196 (22.6 %) patients, and the examination was not standardized, we only prospectively substituted NT-proBNP with LVEF, also leading to loss of significance for TCC (Table 3, Model 3). Due to the difference in time from symptoms to PCI according to levels of TCC, this was added in a model without CRP and NT-proBNP, also leading to a loss of significance (Table S2; Model 6).

**Table 3:** Crude and adjusted Cox regression analysis of the association between above- vs. below-median TCC and total mortality.

	n	Unadjusted HR	95 % CI	*p*-value	Model 1 HR	95 % CI	p-value	Model 2 HR	95 % CI	p-value	Model 3 HR	95 % CI	p-value
Above vs. below median TCC	864	1.650	1.020, 2.671	0.041	1.673	1.014, 2.761	0.044	1.437	0.837, 2.468	0.188	1.244	0.678, 2.283	0.480
Age	864	1.089	1.065, 1.113	<0.001	1.070	1.045, 1.096	<0.001	1.074	1.047, 1.102	<0.001	1.058	1.029, 1.088	<0.001
Male vs. female gender	864	0.376	0.233, 0.609	<0.001	0.554	0.330, 0.932	0.026	0.538	0.316, 0.916	0.022	0.581	0.317, 1.066	0.080
Hypertension	864	2.092	1.309, 3.344	0.002	1.292	0.781, 2.138	0.319	1.355	0.805, 2.279	0.253	1.354	0.756, 2.426	0.308
LDL cholesterol	832	0.603	0.469, 0.774	<0.001	0.680	0.529, 0.875	0.003	0.689	0.528, 0.899	0.006	0.718	0.535, 0.964	0.028
C-reactive protein	862	1.007	1.002, 1.011	0.002				1.000	0.994, 1.006	0.900	1.002	0.996, 1.008	0.464
NT-proBNP	836	1.001	1.001, 1.001	<0.001				1.000	1.000, 1.001	0.133			
LVEF	668	0.940	0.915, 0.967	<0.001							0.961	0.934, 0.989	0.007
Peak TnT	864	1.029	0.989, 1.071	0.153									

Hazard ratios calculated using Cox proportional hazard regression models. Model 1 is adjusted for age, gender, hypertension and LDL cholesterol. Model 2 is adjusted for age, gender, hypertension, LDL cholesterol, C-reactive protein and NT-proBNP. Model 3 is adjusted for age, gender, hypertension, LDL cholesterol, C-reactive protein and LVEF. The HR of continuous variables refers to per year increase for age, per unit increase for the biochemical variables and per percentage point increase for LVEF. HR: hazard ratio; CI: confidence interval; TCC: terminal complement complex; LDL low-density lipoprotein; NT-proBNP: NT-pro brain natriuretic peptide; LVEF: left ventricular ejection fraction; TnT: Troponin T.

### 3.4 Predictive value of combining TCC and NETs markers

When examining patients in the highest quartile of both TCC *and* dsDNA (*n* = 68), they did not have an increased risk of the composite endpoint compared to patients in Q1–3 of TCC *and* dsDNA (Table S3). The risk of total mortality for patients in Q4 of dsDNA and Q1–3 of TCC was similar to those in Q4 of both dsDNA *and* TCC (Table 4).

**Table 4:** Cox regression of the risk of total mortality based on quartiles of TCC and dsDNA, adjusted for age and gender.

	n (endpoints)	HR	95 % CI	p-Value
Q1–3 dsDNA and Q1–3 TCC	523 (28)	Ref.	–	–
Q4 TCC and Q1–3 dsDNA	142 (11)	1.488	0.739, 2.995	0.266
Q4 dsDNA and Q1–3 TCC	119 (16)	3.775	2.020, 7.953	<0.001
Q4 dsDNA and Q4 TCC	68 (12)	3.440	1.741, 6.798	<0.001

HR: hazard ratio; CI: confidence interval; Q: quartile; dsDNA: double-stranded DNA; TCC, terminal complement complex.

In ROC curve analysis of total mortality, the area under the curve (AUC) was 0.549 (95 % CI: [0.472, 0.625]) for TCC alone and 0.653 (95 % CI: [0.579, 0.720]) for dsDNA alone. When combining TCC and dsDNA, the AUC remained virtually the same as for dsDNA alone (AUC: 0.660, 95 % CI: [0.590, 0.730]). Combining TCC with MPO-DNA or CitH3 in ROC curve analysis did not add to the prognostic value of TCC alone (data not shown).

## 4 Discussion

In this STEMI population, complement activation assessed by TCC was not associated with clinical endpoints, defined as a composite of reinfarction, stroke, unscheduled revascularization >3 months after the index infarction, rehospitalization for heart failure, or death. However, above-median levels of TCC were to a certain degree associated with total mortality after a median of 4.6 years. Combined high levels of TCC and the NETs marker dsDNA were associated with total mortality, but this combination did not increase the prognostic value substantially compared to dsDNA alone, which we previously have demonstrated to associate with mortality in this population [18]. These observations indicate that TCC levels might be of clinical relevance after STEMI.

Complement activation measured by TCC has previously been shown to predict cardiovascular outcomes in patients with STEMI [6]. This could not be confirmed in the present study. One possible explanation could be the time of blood sampling. In our study, blood samples were taken the morning after PCI, while in previous reports, samples were drawn before revascularization. Complement activation products, including TCC, have been shown to deposit in damaged myocardium following myocardial infarction [9]. TCC deposition may be exacerbated by revascularization and IR injury, and circulating TCC measured *after* revascularization might thus imprecisely reflect myocardial damage as a larger proportion of TCC is probably bound to the myocardium. This may partly explain why TCC did not predict the composite endpoint in the present study.

Although not predictive for the composite endpoint, TCC was associated with total mortality, also when adjusting for several traditional and associated covariates. The significance was, however, lost when adjusting for CRP. A strong inflammatory reaction is presumed to be central in both IR injury and the initial myocardial remodeling phase [3]. Some inflammation is crucial as it clears dead cells and forms a scar in the damaged myocardium preventing cardiac rupture. Excessive inflammation however, causes fibrosis that predispose to heart failure [22]. Complement activation might contribute negatively to this balance, and thus associate with total mortality before adjusting for the “general” systemic inflammation. Significance was also lost when adjusting for NT-proBNP. This may be a reflection of NT-proBNP as a marker of myocardial stress and damage, but whether this has resulted from complement mediated IR-injury cannot be determined in our observational study [23].

Complement activation was weakly associated with the NETs markers dsDNA and CitH3, but not MPO-DNA. There is experimental evidence for reciprocal activation between the complement system and NETs [19]. Many complement activation products contribute to NETs release [24], [25], [26], and NETs have been shown to activate the complement system [27], [28]. The weak association in the present study might indicate that the interactions mostly occur within the coronary thrombus or in the coronary circulation, and are thus not detectable in the systemic circulation. Concordant with this, higher levels of NETs markers and complement activation products at the culprit site than in systemic circulation has previously been reported [16], [29], [30], [31]. As both systems are implicated in IR injury [2], [5], [14], [32], activation of both systems may cause a vicious cycle of exacerbated complement activation *and* NETs release, contributing to increased myocardial damage following STEMI. However, we could not show this in the present study, as combining TCC and dsDNA did not increase predictive value compared to dsDNA alone.

## 5 Limitations

As blood was drawn at different time points following STEMI, and biomarker concentrations can change significantly in this time, this is a weakness with the present study. Additionally, significance of the risk conferred by TCC was lost when adjusting for time from symptoms to PCI, indicating that increased myocardial damage might be a confounding factor contributing to both increased TCC and total mortality. It should also be emphasized that deaths included in total mortality included all forms for death, not only cardiovascular mortality. TCC is a well-established marker of complement activation, but the balance between circulating and cell-bound TCC is not constant, and can be influenced by several factors [33]. This might lead to large discrepancies within a patient population, and further complicate the prospect of drawing specific conclusions. Implementation of the NETs markers is also challenging. While all presently utilized markers represent the presence of NETs, they inter-correlate weakly, and each marker poses challenges of its own [34]. dsDNA especially can stem from any nucleated cell, and thus represent general cell damage.

## 6 Conclusions

In this STEMI population, complement activation measured by TCC after revascularization was not associated with a composite of long-term clinical endpoints, but was associated with increased risk of total mortality. However, this association was lost when adjusting for potential confounders. Despite a high mortality rate in patients with high levels of both TCC and the NETs marker dsDNA, combining the two did not increase the prognostic value substantially compared to dsDNA alone.

The following are the supplementary data related to this article.

**Fig. S1a** Survival free of events according to quartiles of TCC. Q: quartile; TCC: terminal complement complex.
**Fig. S1b** Survival curves according to quartiles of TCC. Q: quartile; TCC: terminal complement complex. Fig. S1

Supplementary tables Image 1

## Credit authorship contribution statement

KEK performed laboratory and statistical analyses, contributed to interpretation of results and drafted the manuscript. MSL contributed to the interpretation of data. HA and TT, contributed to discussion of results and intellectual content of the manuscript. SH and GØA contributed to the main study design and intellectual content of the manuscript. IS and RH contributed to the sub-study design, interpretation of the results and the intellectual content of the manuscript. All authors read and approved the final manuscript.

## Declaration of competing interest

The authors declare that they have no known competing financial interests or personal relationships that could have appeared to influence the work reported in this paper.
