# Supplementary figures and images for: Complement activation in association with clinical outcomes in ST-elevation myocardial infarction
Source: Am Heart J Plus. 2022 Nov 19;24:100228. doi: 10.1016/j.ahjo.2022.100228 (PMC10978422; doi:10.1016/j.ahjo.2022.100228)

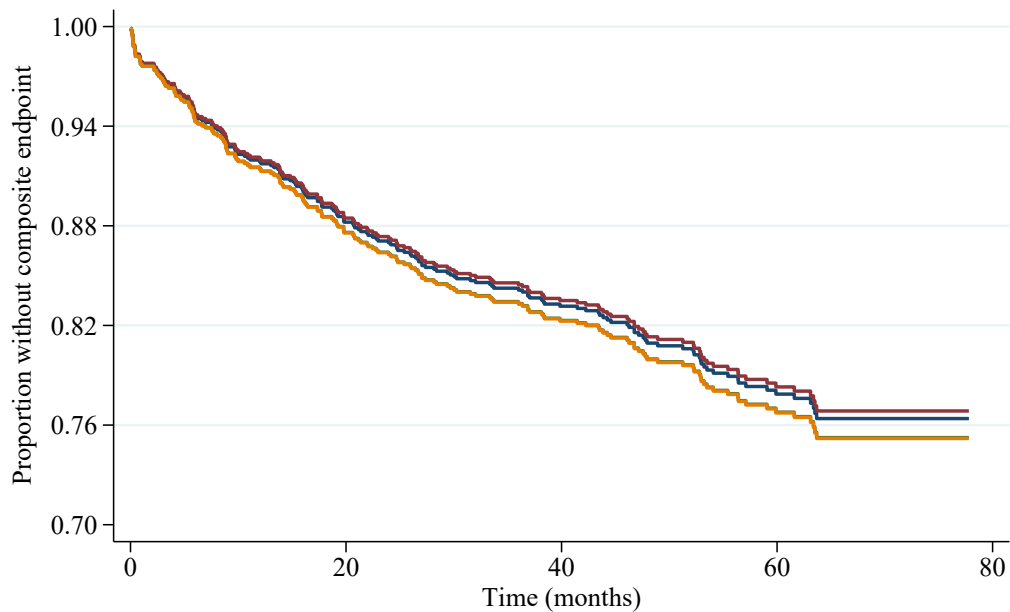

Number at risk

|    |     |     |     |    |   |
|----|-----|-----|-----|----|---|
| Q1 | 215 | 193 | 163 | 80 | 0 |
| Q2 | 217 | 187 | 152 | 81 | 0 |
| Q3 | 216 | 190 | 152 | 75 | 0 |
| Q4 | 216 | 190 | 156 | 87 | 0 |

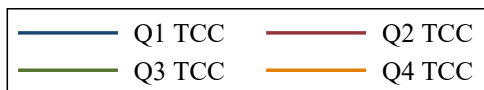

Supplement: Supplementary file 1 — Fig. S1a Survival free of events according to quartiles of TCC. Q: quartile; TCC: terminal complement complex. Fig. S1b Survival curves according to quartiles of TCC. Q: quartile; TCC: terminal complement complex. [file mmc1.pdf]

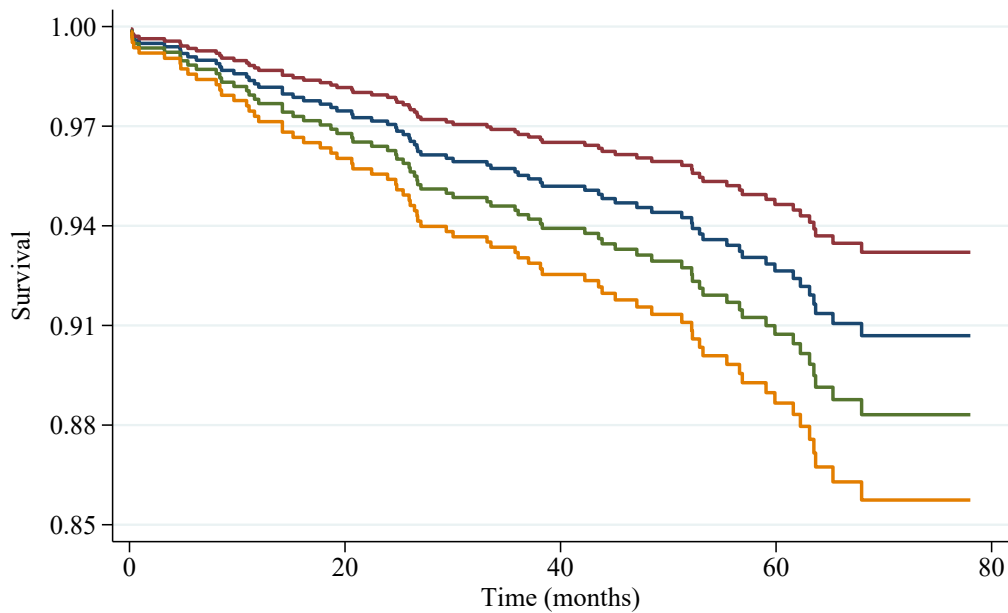

Number at risk

|    |     |     |     |     |   |
|----|-----|-----|-----|-----|---|
| Q1 | 215 | 209 | 187 | 101 | 0 |
| Q2 | 217 | 211 | 181 | 96  | 0 |
| Q3 | 216 | 210 | 170 | 91  | 0 |
| Q4 | 216 | 209 | 176 | 101 | 0 |

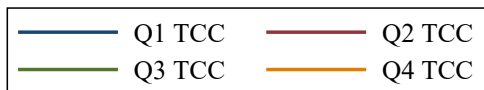

Supplement: Supplementary file 2 — Fig. S1a Survival free of events according to quartiles of TCC. Q: quartile; TCC: terminal complement complex. Fig. S1b Survival curves according to quartiles of TCC. Q: quartile; TCC: terminal complement complex. [file mmc2.pdf]
