# Supplementary material for: Complement activation in association with clinical outcomes in ST-elevation myocardial infarction
Source: Am Heart J Plus. 2022 Nov 19;24:100228. doi: 10.1016/j.ahjo.2022.100228 (PMC10978422; doi:10.1016/j.ahjo.2022.100228)
Supplement: Supplementary file 3 — Supplementary tables [file mmc3.docx]

# Supplementary material

**Table S1** Baseline characteristics of patients according to the composite endpoint

|  | Endpoint + (n=184) | Endpoint – (n=680) | p-value |  |
| --- | --- | --- | --- | --- |
| Age, mean (range) | 63.5 (29-94) | 59.9 (24-90) | **<0.001** |  |
| Female gender | 41 (22.3) | 132 (19.4) | 0.388 |  |
| Smoking | 85 (46.2) | 329 (48.5) | 0.575 |  |
| Hypertension | 68 (37.0) | 215 (31.6) | 0.171 |  |
| Diabetes | 28 (15.2) | 77 (11.3) | 0.152 |  |
| BMI, kg/m^2^ | 26.5 (24.3, 29.4) | 26.6 (24.3, 29.2) | 0.991 |  |
| eGFR | 91.4 (80.8, 101.9) | 96.7 (87.5, 104.0) | **<0.001** |  |
| Total leukocyte count x 10^9^/L | 10.85 (8.4, 14.0) | 10.60 (8.78, 13.00) | 0.369 |  |
| Platelet count x 10^9^/L | 228 (190, 271) | 217 (185, 263) | 0.112 |  |
| Total cholesterol, mmol/L | 4.72 ±1.23 | 4.98 ±2.10 | **0.032** |  |
| LDL-cholesterol, mmol/L | 3.11 ±1.15 | 3.28 ±0.98 | **0.034** |  |
| HDL-cholesterol, mmol/L | 1.15 ±0.57 | 1.12 ±0.34 | 0.549 |  |
| Triglycerides, mmol/L | 1.38 ±0.80 | 1.46 ±0.90 | 0.174 |  |
| Fasting glucose, mmol/L | 5.9 (5.4, 6.8) | 5.7 (5.2, 6.5) | **0.016** |  |
| C-reactive protein, mg/L | 15.02 (7.19, 40.39) | 13.16 (6.95, 29.22) | 0.292 |  |
| Peak TnT ng/L | 3875 (1425, 7493) | 3815 (1735, 7018) | 0.695 |  |
| NT-proBNP pg/mL | 50 (13, 178) | 26 (10, 103) | **0.001** |  |
| LVEF ≤ 40% | 35 (26.3) | 98 (18.3) | **0.039** |  |
| Symptom to PCI time, hours | 4 (3, 7) | 4 (3, 6) | 0.390 |  |
| PCI to blood sampling, hours | 19 (13, 23) | 18 (12, 22) | 0.387 |  |
| **Previous CVD** |  |  |  |  |
| Myocardial infarction | 19 (10.3) | 76 (11.2) | 0.739 |  |
| PCI | 36 (19.6) | 58 (8.5) | **<0.001** |  |
| Heart failure | 8 (4.3) | 9 (1.3) | **0.007** |  |
| Stroke | 15 (8.2) | 22 (3.2) | **0.003** |  |
| **Medication:** |  |  |  |  |
| Single or DAPT | 58 (31.5) | 137 (20.1) | **0.001** |  |
| Statins | 52 (28.3) | 138 (20.3) | **0.021** |  |
| Beta blockers | 45 (24.5) | 116 (17.1) | 0.065 |  |
| ACEi/ARB | 57 (31.0) | 149 (21.9) | **0.034** |  |

Values are given as mean (±SD), median (25th, 75th percentiles) or numbers (%) as appropriate. BMI: body mass index; eGFR: estimated glomerular filtration rate; LDL: low-density lipoprotein; HDL: high-density lipoprotein; TnT: troponin T; NT-proBNP: NT-pro brain natriuretic peptide; LVEF: left ventricular ejection fraction; CVD: cardiovascular disease; PCI: percutaneous coronary intervention; DAPT: dual antiplatelet therapy; ACEi: angiotensin converting enzyme inhibitor; ARB: angiotensin II receptor blocker.

**Table S2** Crude and adjusted Cox regression analysis of the association between TCC and all-cause mortality.

|  | n | Unadjusted HR | 95% CI | p-value | Model 4 HR | 95% CI | p-value | Model 5 HR | 95% CI | p-value | Model 6 HR | 95% CI | p-value |
| --- | --- | --- | --- | --- | --- | --- | --- | --- | --- | --- | --- | --- | --- |
| Above vs. below median TCC | 864 | 1.650 | 1.020, 2.671 | **0.041** | 1.429 | 0.885, 2.515 | 0.133 | 1.547 | 0.916, 2.613 | 0.103 | 1.407 | 0.827, 2.395 | 0.208 |
| Age | 864 | 1.089 | 1.065, 1.113 | **<0.001** | 1.072 | 1.046, 1.100 | **<0.001** | 1.070 | 1.045, 1.096 | <0.001 | 1.074 | 1.046, 1.102 | **<0.001** |
| Male vs. female gender | 864 | 0.376 | 0.233, 0.609 | **<0.001** | 0.546 | 0.322, 0.926 | **0.025** | 0.551 | 0.326, 0.929 | 0.025 | 0.526 | 0.299, 0.923 | **0.025** |
| Hypertension | 864 | 2.092 | 1.309, 3.344 | **0.002** | 1.325 | 0.791, 2.220 | 0.285 | 1.326 | 0.799, 2.200 | 0.275 | 1.315 | 0.764, 2.265 | 0.323 |
| LDL cholesterol | 832 | 0.603 | 0.469, 0.774 | **<0.001** | 0.700 | 0.540, 0.909 | **0.007** | 0.681 | 0.526, 0.880 | 0.003 | 0.638 | 0.487, 0.836 | **0.001** |
| NT-proBNP | 836 | 1.001 | 1.001, 1.001 | **<0.001** | 1.000 | 1.000, 1.001 | 0.087 |  |  |  |  |  |  |
| C-reactive protein | 862 | 1.007 | 1.002, 1.011 | **0.002** |  |  |  | 1.002 | 0.997, 1.007 | 0.458 |  |  |  |
| Time from symptoms to PCI | 779 | 1.010 | 0.988, 1.033 | 0.385 |  |  |  |  |  |  | 1.006 | 0.980, 1.033 | 0.671 |
| LVEF | 668 | 0.940 | 0.915, 0.967 | **<0.001** |  |  |  |  |  |  |  |  |  |
| Peak TnT | 864 | 1.029 | 0.989, 1.071 | 0.153 |  |  |  |  |  |  |  |  |  |

Model 4 is adjusted for age, gender, hypertension LDL cholesterol and NT-proBNP, Model 5 is adjusted for age, gender, hypertension LDL cholesterol and C-reactive protein, Model 5 is adjusted for for age, gender, hypertension LDL cholesterol and time from symptoms to PCI. The HR of continuous variables refers to per year increase for age, per unit increase for the biochemical variables, per percentage point increase for LVEF and per hour increase for time from symptoms to PCI. HR: hazard ratio; TCC: terminal complement complex; LDL low-density lipoprotein; NT-proBNP: NT-pro brain natriuretic peptide; PCI: percutaneous coronary intervention; LVEF: left ventricular ejection fraction; TnT: Troponin T.

**Table S3** Cox regression analyses of the risk of suffering a composite endpoint in groups based on quartiles of TCC and dsDNA, adjusted for age and gender.

|  | n (endpoints) | HR | 95% CI | p-value |
| --- | --- | --- | --- | --- |
| Q1-3 dsDNA and Q1-3 TCC | 523 (97) | Ref. | - | - |
| Q4 TCC and Q1-3 dsDNA | 142 (28) | 1.055 | 0.693, 1.606 | 0.804 |
| Q4 dsDNA and Q1-3 TCC | 119 (35) | 1.899 | 1.288, 2.799 | **0.001** |
| Q4 dsDNA and TCC | 68 (19) | 1.523 | 0.931, 2.493 | 0.094 |

HR: hazard ratio; CI: confidence interval; Q: quartile; dsDNA: double-stranded DNA; TCC. terminal complement complex.
